# Understanding delayed access to antenatal care: a qualitative interview study

**DOI:** 10.1186/1471-2393-14-207

**Published:** 2014-06-16

**Authors:** Rosalind Haddrill, Georgina L Jones, Caroline A Mitchell, Dilly OC Anumba

**Affiliations:** 1 School of Health and Related Research, University of Sheffield, Regent Court, 30 Regent Street, Sheffield S1 4DA, UK; 2 Academic Unit of Primary Medical Care, Samuel Fox House, Northern General Hospital, University of Sheffield, Herries Road, Sheffield S5 7AU, UK; 3 Academic Unit of Reproductive and Developmental Medicine, University of Sheffield, The Jessop Wing, Tree Root Walk, Sheffield S10 2SF, UK

**Keywords:** Pregnancy, Antenatal care, Access, Late booking, Qualitative study

## Abstract

**Background:**

Delayed access to antenatal care ('late booking’) has been linked to increased maternal and fetal mortality and morbidity. The aim of this qualitative study was to understand why some women are late to access antenatal care.

**Methods:**

27 women presenting after 19 completed weeks gestation for their first hospital booking appointment were interviewed, using a semi-structured format, in community and maternity hospital settings in South Yorkshire, United Kingdom. Interviews were transcribed verbatim and entered onto NVivo 8 software. An interdisciplinary, iterative, thematic analysis was undertaken.

**Results:**

The late booking women were diverse in terms of: age (15–37 years); parity (0–4); socioeconomic status; educational attainment and ethnicity. Three key themes relating to late booking were identified from our data: 1) '**not knowing**’: realisation (absence of classic symptoms, misinterpretation); belief (age, subfertility, using contraception, lay hindrance); 2) '**knowing**’: avoidance (ambivalence, fear, self-care); postponement (fear, location, not valuing care, self-care); and 3) '**delayed**’ (professional and system failures, knowledge/empowerment issues).

**Conclusions:**

Whilst vulnerable groups are strongly represented in this study, women do not always fit a socio-cultural stereotype of a 'late booker’. We report a new taxonomy of more complex reasons for late antenatal booking than the prevalent concepts of denial, concealment and disadvantage. Explanatory sub-themes are also discussed, which relate to psychological, empowerment and socio-cultural factors. These include poor reproductive health knowledge and delayed recognition of pregnancy, the influence of a pregnancy 'mindset’ and previous pregnancy experience, and the perceived value of antenatal care. The study also highlights deficiencies in early pregnancy diagnosis and service organisation. These issues should be considered by practitioners and service commissioners in order to promote timely antenatal care for all women.

## Background

Antenatal care is widely acknowledged as contributing to improved pregnancy outcomes, with delayed access ('late booking’) linked to increased maternal, fetal and infant mortality and morbidity. The previous five United Kingdom (UK) Maternal Mortality reports have identified late booking as a significant risk factor for maternal death for all women, and particularly black and minority ethnic women [1,2]. Different definitions of late booking exist, from 16–22 weeks gestation. The UK prevalence of booking after 18–20 weeks gestation is reported at between 2.8% and 16%, similar to rates reported for continental Europe and the United States of America (USA), but with some studies showing significant regional variation [3-9]. UK guidance recommends that all pregnant women should have had their first antenatal appointment with a midwife by 10–12 completed weeks of pregnancy, in order to identify and respond to clinical and social risk factors, and that all antenatal screening should be completed before 21 weeks gestation [2,10,11].

Observational studies, mostly from outside the UK, have suggested that 'late bookers’ for antenatal care are typically from socially excluded groups; ethnicity in particular, but also young age, low income and educational level, lack of support and substance misuse have been found to be common characteristics in this group of women [3,4,7,12-14]. However, little explanatory qualitative research concerning women’s attitudes towards access to, and initiation of, antenatal care has been undertaken. A meta-analysis [15] and a Command paper published by the Department of Health (DoH) [16] identified this as an important gap in the literature and recommended further research in this area.

A subsequent DoH study surveyed a range of 'hard to reach’ groups, identifying a number of interrelated barriers, which delayed, curtailed or even prevented access to antenatal care [17]. Callaghan *et al.*, in a London-based qualitative study, similarly found varied and complex reasons for non-attendance antenatally [18]. Downe *et al’s*[19] meta-synthesis of barriers to antenatal care concludes that the reasons for late, infrequent or non-attendance at antenatal services in the UK remain to be fully evaluated, in order for appropriate interventions to be developed. Consequently, we aimed to carry out a qualitative study with a broad range of late booking women, with our objective being to gain a deeper understanding of the reasons why some women present late for antenatal care. This article details the method and findings of the study and discusses these in relation to previous research on late booking.

## Methods

Semi-structured individual interviewing with late booking pregnant women was chosen to provide a flexible and personal method of data collection, which had the potential to generate rich detail and insight. The topic guide for the interviews was developed after a literature review and discussion within a multidisciplinary group comprising of an academic social scientist, obstetrician, midwives and a General Practitioner (GP). It is summarised in Table 1. Late booking was defined as first hospital antenatal attendance at 20 or more weeks gestation. This is because although recent UK antenatal guidance for pregnant women with complex social factors [20] identifies the limit of early booking as 12^+6^ weeks (and the Euro-Peristat definition [21] is by the end of the first trimester: 14 weeks), late booking is defined as 20 weeks. It was recognised that women booking at 13 weeks gestation are likely to demonstrate significantly different reasons and attitudes towards their care than a woman booking at a more advanced gestation such as 20 weeks and beyond. The gestation was chosen to maximise the number of women who had purposefully chosen to delay the initiation of care, rather than as a result of the late discovery of pregnancy.

**Table 1:** An overview of the semi-structured interview guide

**Theme**	**Selected questions**
**Discovery of pregnancy**	Tell me about your experience of finding out you were pregnant.
**Acceptance of pregnancy**	Are there any reasons why you have attended later than usual for antenatal booking?
	Are there any factors, which may have prompted or helped you see a doctor or a midwife earlier in your pregnancy, or attend earlier for your booking visit?
**Knowledge of 'booking’**	What is your understanding of the first antenatal booking visit? Are you aware that it is recommended between the 8^th^ and 12^th^ weeks of pregnancy?
	When ideally would you first like to see a midwife or a doctor during pregnancy, and why?
**General health/support**	Do you often visit your family doctor (GP)?
	Have you sought advice or support from other sources?

Women who had received antenatal care, other than an initial referral appointment with their community midwife, prior to this hospital attendance, were excluded. Attendance at hospital, rather than this initial appointment, was chosen to examine whether external as well as personal factors were influential. In Sheffield, 6% of pregnant women booked after 19 weeks gestation in 2009–2010, though this figure was higher amongst women aged under 20 (8.7%) [22]. In accordance with other qualitative studies with socially excluded groups we anticipated that we would need to interview around 25 women. A purposive recruitment strategy was theoretically informed by the literature review: we aimed to recruit a maximum variety sample of women and to continue to interview until no new themes emerged [23]. Ethical Approval for the study was obtained from the North Sheffield Ethics Committee (reference 05/Q2308/153).

The individual semi-structured interview format enabled participants to express their views on the topic and generated additional areas of discussion. It developed during the study through the iterative processes of simultaneous data collection and analysis, and constant comparison. The interviews were undertaken in community settings and hospital antenatal clinics and wards. We used a number of sampling strategies in order to identify women for the study. Firstly, antenatal clinic notes and referral letters were reviewed to identify any woman attending at more than 19 weeks gestation; these women were then followed up by the research midwife. Secondly, we attempted to obtain a diverse (maximum variety) sample of late booking women that included 'at risk ' groups identified from the literature, for example teenagers, recent migrants, women with substance misuse problems, women with learning disabilities, and overall, as far as was possible, women from a variety of other social and occupational backgrounds. This purposive sampling strategy involved intensive networking with key healthcare practitioners including community and hospital midwives, specialist midwives and doctors (for example substance misuse and teenage pregnancy clinics) involved in the care of potential late booking women.

We continued to interview women until no new themes emerged, whilst ensuring diversity according to the above theoretically informed criteria. The recruitment process is illustrated in Table 2. At the first hospital antenatal clinic appointment, potential participants were given written information about the study and gave verbal consent for us to contact them to arrange an interview, at a time and in a location of their choice. There was no funding for interpreters, however a few women whose first language was not English were recruited, where they were able to understand the project fully and give written informed consent to participate. Particular care was taken when seeking consent from more vulnerable women, in particular the four teenagers aged less than 18 years and the three women with learning disabilities, to ensure that the study and their voluntary participation was clearly explained and understood, in some cases in consultation with supporting adults. Attention was also made to the interview format and location for these women to try to put them at ease. No financial inducements were made to participants, although reimbursements for any travel expenses incurred were offered.

**Table 2:** The recruitment process

*Women recruited in South Yorkshire maternity unit, September 2006 – July 2008*	
*Women attending for first antenatal (booking) appointment with gestation (confirmed by USS) > 19*^*+6*^*/40.*	
*Potential candidates identified from referral letters by research midwife and through discussion with antenatal clinic midwives and specialist midwifery teams.*	
**Eligible women given information pack and asked for initial consent for research midwife to contact (n = 83)**	
	Not meeting entry criteria (n = 3)
**Contacted by telephone and/or post by research midwife (n = 80)**	
	Unable to contact by telephone/post (n = 39)
**Contacted by phone and interview arranged at home or next antenatal appointment (n = 41)**	
	Did not attend for interview and unable to contact by telephone/post (n = 10)
	Declined to be interviewed (n = 4)
**Interviewed at home, community children’s centre or hospital (n = 27)**	

Prior to each interview, informed consent was obtained and demographic and postcode data were recorded. Interviews were tape recorded, transcribed verbatim and checked for accuracy. A self-conscious thematic analysis of data was undertaken by the research team, using the methods outlined by Braun and Clarke [24]. NVivo8 (QSR International, Burlington MA.) software was used to organise the transcribed data. Consideration of reflexivity and potential researcher bias were important, as RH is a registered midwife and PhD student, GLJ is an academic social scientist, CM is an academic GP and DOA is an academic consultant specialist in fetomaternal medicine. All the interviews were carried out by RH who had undergone training in qualitative interviewing as part of a research post and she was not involved in the clinical care of any of the women. In addition to independent analysis of transcribed data (RH, GJ, CM), interdisciplinary analysis meetings were held that included critical appraisal of the literature, systematic data and coding framework verification and challenging of interpretive analysis.

## Results

Twenty-seven women were interviewed. Their demographic details are summarised in Table 3. Twenty-one women were White British, whilst six were from other ethnic groups. The women reported a wide range of occupations and educational levels; many lived in neighbourhoods with high deprivation indices.

**Table 3:** Participant characteristics (n = 27)

**Age at interview**	
Mean age (range)	26 (15–37)
**Marital status**	
Married	10 (37%)
Cohabiting	7 (26%)
Single	10 (37%)
**Ethnic origin**	
White	21 (77.8%)
Mixed White/Caribbean	1 (3.7%)
Pakistani	1 (3.7%)
White European (Dagestani)	1 (3.7%)
Black African (Eritrean)	1 (3.7%)
Other (Mexican, Saudi)	2 (7.4%)
**Parity** (2 women had previous children removed and placed for adoption)	
0	14 (52%)
1	7 (26%)
2	2 (7%)
3 or more	4 (15%)
**Educational level** (3 women had learning disabilities)	
Up to 16 years (secondary school)	13 (48%)
Further education	7 (26%)
Higher/university education	5 (19%)
Unknown	2 (7%)
**Occupation**^**1**^	
None	3 (11%)
Student	7 (26%)
Housewife	6 (22%)
Elementary occupations	1 (4%)
Personal service occupations	5 (18%)
Sales and customer service	2 (8%)
Professional occupations/managers	3 (11%)
**Deprivation ranking of home address (2010)**^**2**^	
Living in lowest 5% of English neighbourhoods	9 (33%)
Living in lowest 20% of English neighbourhoods	15 (56%)
Living in lowest 50% of English neighbourhoods	20 (74%)

^1^Occupation from: Standard Occupational Classification 2000 (SOC2000), accessed from: http://www.ons.gov.uk/ons/guide-method/classifications/archived-standard-classifications/standard-occupational-classification-2000/index.html.

^2^English Index of Multiple Deprivation Score (2010) for Lower Layer Super Output Areas (LSOAs) accessed from: http://www.neighbourhood.statistics.gov.uk/dissemination/.

The interviews identified a variety of often interrelated personal and service organisational reasons for not accessing antenatal care earlier. These included not recognising the pregnancy, recognising but avoiding or postponing antenatal care, and practical difficulties resulting in delay such as their location, administrative errors and the failure of healthcare professionals to diagnose pregnancy and/or expedite care for late bookers. A taxonomy of reasons for late presentation derived from the thematic analysis of the data is presented in Table 4.

**Table 4:** Summary of women’s themes for late booking

**Not knowing**	**Realisation**	Delayed confirmation of pregnancy	Lack of cardinal symptoms of pregnancy	
			Symptoms masked by irregular periods	
		Women’s misinterpretation/misdiagnosis of symptoms	Attributed to other life event	'Mindset’
			Attributed to past/current medical condition	
			Lack of reproductive knowledge/pregnancy experience	Learning disabilities
		Influence of others	Lay hindrance	
			Rejection of lay influence	
	**Belief**	Age affecting fertility		
		Past illness affecting fertility		
		Using contraception		
		Not planning, expecting to be pregnant	'Mindset’	
			Ambivalence	
**Knowing**	**Avoidance**	Fear and ambivalence	Delay in confirming pregnancy	
			Fear of 'consequences’ of pregnancy	Fear of removal of child
				Fear of stigma, judgement
		Coping mechanisms	Denial, concealment	In control of decision
			Antenatal self-care	Using knowledge, experience
	**Postponement**	Lack of perceived value of antenatal care	(Good) past experience of pregnancy	Previous concealed pregnancy/late booking
			Feeling well	
			Acceptance	Fatalism, religion
			Antenatal self-care	
			'On the move’	Portability and timing of care
				Waiting until 'home’/in place of trust/safety
				Other priorities: no hurry’
				Practical difficulties accessing care
		Fear and ambivalence	Fear of 'consequences’ of pregnancy	Fear of judgement, reaction
				Convenience
				Denial
				Coping with another child
				Previous traumatic childbirth
				Fear of blood tests
			Considering termination	Indecision resulting in delay
				Unplanned, unwanted pregnancy
		Pressure to have a termination	Protecting the pregnancy	In control of decision
**Delayed**	**Professional failures in primary care**	Misdiagnosis, misinformation		
		Mis-estimation of gestation		
	**System failures**	Delay in referral process/scheduling of appointments		
		Lost appointments		
	**Women’s knowledge and empowerment issues**	Not challenging the system	Lack of knowledge of the antenatal care system	'Mindset’
		Rationalising the delay	Trusting healthcare professionals	
			Feeling well, care not important	
			Influence of family and friends	

### “Not Knowing”

#### Realisation

Many of the women interviewed had said they had not known they were pregnant for weeks or sometimes months, which had delayed them accessing care. These were divided into women who either had not noticed any of the 'cardinal’ symptoms of pregnancy (e.g. nausea, vomiting and amenorrhoea), or those who had symptoms but did not recognise them as pregnancy. Reported 'normal’ cycle irregularity masked the ability of some women to perceive classic pregnancy symptoms early enough to access antenatal care:

*“To be honest, my periods aren’t regular so I didn’t know how many weeks I was. I can go without periods for 6 months… And because of my periods I suppose it took a while before I knew I was pregnant for definite”.* (#9, Gravida 3 Para 2)

Multiparous women had also attributed symptoms such as amenorrhoea, nausea or weight change to life events:

*“I had a bleed and I thought I’d just had a period. We were opening this pub and it was just really stressful and I thought I was feeling sick because we weren’t eating. We were working from 10 in the morning until 2 or 3 at night. So I just thought I was run down”.* (#6, G2 P1)

Other women, in retrospect, recognised that they had experienced pregnancy symptoms but had misinterpreted these due to a lack of knowledge or experience. Key groups for whom a lack of reproductive knowledge was the main reported reason were those women with learning disabilities and young women. In these instances, it was often family members or close friends who noticed the pregnancy before the woman herself:

*“I found movements moving about in my stomach and I wondered what it was… I didn’t have any sickness or anything like that, so I went to see my GP, he examined me and said I could be like 6 months pregnant”.* (#2, G1P0)

*“My dad had an idea that I could be pregnant because I was a bit swollen. He said “you’re pregnant”. I said “I haven’t got an idea”. He said “I think you ought to go and get checked”. So a fortnight later I went and checked, I went back home and said “Dad, you’re right, I am pregnant”. He went “I told you I was right didn’t I”.* (#7, G1P0, learning disabilities)

We also identified lay hindrance as a barrier. Some women reported that family, friends and partners had also attributed the classical pregnancy symptoms to another reason, most evident in relation to symptoms of nausea and vomiting:

*“We all just used to be sat there and I'd go to the toilet and M used to say 'she’s got an upset stomach’. That’s all she used to say, that’s all we put it down to, just an upset stomach, we didn’t think of anything else”.* (#28, G2P1, learning disabilities)

#### Belief

17 of the 27 women interviewed stated that they had not planned to become pregnant, for another 6 this was implied and others suggested that their pregnancy was intended but mistimed. These women did not have a pregnancy 'mindset’, so were not expecting the symptoms of pregnancy, thus leading to a delay in diagnosis and initiation of antenatal care. For example, some women in their thirties had assumed age would more significantly adversely affect their fertility than was actually the case, a belief which in one instance was reinforced by their general practitioner’s initial misdiagnosis:

*“When they (the GPs) said 'I was going through the change’ I thought 'well could I be’ because at 37 I thought 'well I might be’ because you hear women go through it earlier than I did and I think I got that into my head more than anything and I never contemplated that I was pregnant”.* (#13, G5P4)

Other women reported that they didn’t believe they could be pregnant because they had been ill recently or had existing medical conditions associated with sub- or infertility, such as polycystic ovarian syndrome. Contraceptive failure was also a significant factor:

*“Last time, it was just weird because I went for the Depo and they told me I was 25 weeks pregnant. I got caught on the Depo and I didn’t know that, and I took the pills and I got caught on the contraceptive pill this time… and I didn’t know with this one”.* (#28, G2P1, learning disabilities)

In addition to explaining their reasons for late booking, women reflected on the experience. Most of the women interviewed, including young women and primigravidas, were aware of the optimum time to access antenatal care and were clear that they would have booked early, if circumstances had been different. For example, many of the 'not knowing’ women expressed regret at not accessing care earlier and acknowledged the desirability of attending for early antenatal care:

*“If I had known I would have come virtually the first couple of weeks I knew, if you know what I mean, but as I say, I had no clue, no idea. I would have gone straightaway, yes, I would advise anybody to do that straightaway”.* (#12, G5P4)

Many women expressed feelings of guilt at accessing care late; particularly in terms of the negative consequences for their unborn baby that could have arisen from missed screening and not following recommended dietary and lifestyle changes.

*“I missed out on folic acid; I regret that very much. I took it with my other children. In some ways I think I let this baby down, I didn't give it what the others had… I feel guilty about that”.* (#8, G3P2)

This was not a universal response however. Some women who had not known they were pregnant were pleased to have 'missed’ part of the pregnancy: impatience for the pregnancy to be over and their baby to arrive was expressed by several women and their partners; as one woman described

*“It was really good because I thought I won’t have to wait as long, because 9 months is long. Even now, I feel like it’s been such a long time”.* (#16, G1P0)

#### “Knowing”

The study identified a second large group of women who knew that they were pregnant but did not access early care. There were three key themes amongst these women: avoidance, postponement and being delayed by others, with avoidance and postponement themes emerging from 14 of the 27 interviews. Whereas women avoiding care had made no plans to access care, women who postponed their care intended to access care 'at some point’ in the future. Avoidance in the study reflected a woman’s refusal to consider the pregnancy and its consequences. In contrast postponement reflected a period of ambivalence about and evaluation of the pregnancy, as women considered their choices and priorities.

#### Avoidance

Two key themes were identified for why women wanted to avoid antenatal care. Firstly, some women were fearful of the social consequences of the pregnancy i.e. removal of the baby by social services, or the response and judgement from the baby’s father, their family and peers; particularly teenagers who feared the stigma and negative stereotypes that exist surrounding teenage pregnancy.

*“I knew you had to go for all the tests and things like that, but I just couldn’t go. Because I was only 17, I just thought I couldn’t tell anyone, thought people would look at me like…I don’t know, just like I was, irresponsible … I didn’t want to think about it. So I thought I’d just put it to the back of my mind”.* (#8, G1P0)

Secondly, some women, especially the most vulnerable groups such as substance misusing women and those with learning disabilities, described significant ambivalence towards the pregnancy.

*“I had an idea about 2 months before, but I did a pregnancy test and that didn’t work, It didn’t say negative or positive, just no result came up. So then I just put it to the back of my mind, and my mum mentioned it again in a little while and I did another pregnancy test and that worked.* (#15, G1P0, on methadone)

#### Postponement

This group of women did not access antenatal care on time but always intended to do so 'at some point’. For example, some well women chose to seek care at a time “convenient” for them. For many, a good past experience of pregnancy influenced their decision to postpone it, based on the premise that antenatal care was only needed if they felt unwell.

*No, they’ve all been normal thank God and I think if there were any previous problems with them I would have probably found out but I just felt healthy, I felt OK you know, I just felt normal basically and I suddenly saw my belly getting a bit bigger and my clothes weren’t fitting as much”.* (#12, G4P3)

Some women postponed access because of initial ambivalence and because they initially planned to terminate the pregnancy, only 'booking’ when they decided to continue with the pregnancy. Others delayed accessing antenatal care because of their religious belief that antenatal screening for fetal abnormality was unimportant:

*“We are Muslims so we are not allowed to have an abortion. After 40 days from the pregnancy it’s not allowed for you, and before the 40 days you should have some serious problem like your heart’s not good or the baby is very damaged. So it’s not just I don’t want it, because I have already 1 child, so I didn’t think about that at all. I have to accept that really and thank God for it”.* (#19, G2P1)

An intuitive process of 'do it yourself antenatal care’ was reported by some women which included self-checks and active self-care, in order to promote and monitor the healthy progress of their pregnancy, until they felt able to access care.

*“I knew quite a bit anyway I kept referring to my books and just checking and thinking 'oh yes it’s alright’ so there was nothing bad. Bits I’d got from college and things like that, so obviously I was thinking I can feel this so I don’t think there’s anything wrong”.* (#8, G1P0)

*“I’d done everything that I could possibly do myself because obviously with having A, I knew what you could eat, what you couldn’t eat, this that and other, so I followed everything religiously, took my Pregnacare every single day, made sure I drank plenty, had plenty of rest, so I carried out what I knew, but obviously I’d had no checks to make sure everything was progressing alright, I’d had movement, I noted down when I’d had movement and things like that, so I’d done all I could”.* (#27, G2P1)

Being 'on the move’ also acted as a barrier. For example, if a woman was returning home from abroad or working elsewhere in the UK, or living in temporary accommodation, they described deferring access to antenatal care until they felt settled in a place of trust and safety. Overall, there was a lack of understanding of the value and/or the 'portability’ of antenatal care.

*“I didn’t know York, I didn’t have any transport when my partner was out at work every day. I didn’t know where buses used to go… But I didn’t want to change my doctor, because I’m going back home to my own house. I wanted to have my baby in [Sheffield], I didn’t want to have my baby in a town that I didn’t know”.* (#6, G2P1)

Fear was again commonly expressed as having influenced their ability to access care. Some women postponed antenatal care as they feared family reactions and how they might cope with the birth of another child. For one woman there was active postponement of her antenatal care until she was 'safe’ from a perceived obligation to have a termination, after a negative response to the pregnancy from the baby’s father.

*“I knew that my husband didn’t really want one, so I kept it to myself for a while… I really just did not want to go through with it* [termination] *and then I just kept putting it off… thinking, I’ve put it off that long, they’re not going to be able to do anything about it and we will have to carry on”.* (#27, G2P1)

Many of the 'knowing’ women also expressed regret at not accessing early care, and demonstrated an understanding of its benefits. However, as one of them observed, their understanding of what was theoretically 'correct’ and desirable in terms of antenatal care might bear little relation to what had happened in their own pregnancy and the choices they had made:

*“I would advise anybody who knew to go. I just don’t take my own advice!”* (#27, G2P1)

### “Delayed”

This group of women had usually been aware from a relatively early stage that they were pregnant and were willing to engage in timely antenatal care. However, due to a combination of reasons they had booked late. There were examples of professional failures, involving GP and/or nurse misdiagnosis, or mis-attribution of pregnancy symptoms to lifestyle or medical causes:

*“I began to feel really sick and really unwell and lo and behold I was pregnant. But I’d gone to the doctors and I’d gone to see the nurse and I’d gone back repeatedly and they said because you’ve stopped drinking, stopped smoking it’s just all the toxins and such like coming out so nothing to worry about”.* (#17, G1P0)

System failures also occurred, for example, letters not being sent and/or received which were typically the result of failings in secondary care. Women experiencing this often had a lack of knowledge of pregnancy and the antenatal care 'system’ and appropriate scheduling, or language difficulties which prevented them from challenging delays. However lack of empowerment was also a key theme: women passively accepted delayed appointments and typically did not challenge health professional misdiagnosis of early pregnancy symptoms. Some women appeared to rationalise the consequent delay as they were well, or were not in a pregnancy mindset to enable focusing on the actual gestation.

*“I kept thinking 'well it seems a long time for me not [to be seen]’… I could have pushed it more at week 15 if I had thought about it, if I had had more knowledge, but I foolishly thought because I had been given the date that that was it - and I asked my midwife and she said it was a bit surprising but not anything to be worried about”.* (#14 G1P0)

## Discussion

Our study identified three key themes, on the basis of which we suggest a taxonomy for understanding why women present late for antenatal care: 'not knowing’; 'knowing’; and being 'delayed’. Explanatory sub-themes relate to individual circumstances, empowerment and socio-cultural factors. These include the recognition of a mother’s own pregnancy and the influence of a pregnancy 'mindset’, the perceived value of antenatal care and the influence of previous pregnancy experience. Other sub-themes include avoidance and postponement strategies and the acceptance of delay. These themes suggest more complexity than the denial, concealment and disadvantage frequently reported, and that whilst vulnerable groups are strongly represented in this cohort, women do not always fit a socio-cultural stereotype of a 'late booker’ [3,4,13-15]. Many themes associated with late booking found in previous studies of marginalised women are evident amongst women across the social, educational and cultural spectrum in this study [19]. However our themes suggest a different emphasis. Particularly there is a greater emphasis on considerations of convenience, relevance and familiarity (leading to the postponement of care), and a lack of a pregnancy 'mindset’ relating to the expectation of becoming pregnant.

### Method

In the conduct of the study we adhered to established quality criteria for qualitative research [23,25]. We acknowledge the challenges of conducting a study about lack of engagement with antenatal care in a population of women stereotypically seen as 'hard to reach’ e.g. vulnerable and socially disadvantaged. However, in order to address this, our recruitment took place over 22 months during which we used snowballing and active engagement with key health and social care practitioners. Achieving as diverse a sample ethnically as we would have wished was not possible, due to the lack of translation support. This, in combination with some women’s reluctance to participate, lengthened the recruitment phase and has inevitably influenced our findings. Additionally, we did not interview women who had received no antenatal care, which would have added another dimension to our results. However,, the overall diversity, size and exclusivity of our sample, within the context of qualitative research, suggests we were largely successful. The study’s iterative methods of data collection and analysis, informed by inductive approaches such as grounded theory, were highly effective at generating rich data about attitudes towards antenatal care. The location of interviews was influential in this respect: women interviewed in their homes, particularly those who had not realised they were pregnant, were generally open and happy to talk in detail and at length about not accessing care. Similarly, women who had experienced professional or system failures were angry or frustrated and keen to tell their story. However women interviewed in hospital, often the women who had concealed their pregnancies, were reluctant to talk about non-access, being quite defensive in their responses, suggesting that they felt that they would be judged and/or condemned by the healthcare 'establishment’ for making the 'wrong’ choices [26].

### Recognising the pregnancy

This study suggests that for some women 'not knowing’ that they are pregnant is a complex mixture of lack of recognition, acknowledgement and acceptance of the signs, symptoms and consequences of pregnancy. It is influenced by many factors, including knowledge and experience of pregnancy, both personally and amongst a woman’s social network, physical and psychological health and the expectation of becoming pregnant. The concept of 'knowing*’* in this context suggests identification and understanding, an acknowledgement of the physical and social consequences of the pregnancy, from the woman and potentially those around her. Few other studies, particularly quantitative studies, have demonstrated the complex relationship of influences and the importance the notion of a 'pregnancy mindset’ plays.

Women’s apparent poor knowledge and awareness of pregnancy, particularly younger women and women with learning disabilities, and their failure to recognise many early signs and symptoms, were common themes in this study and have been widely reported [19]. Some misinterpreted pregnancy symptoms and attributed even multiple symptoms to causes other than pregnancy, especially when their perceived likelihood of becoming pregnant was low, for example for age, health or contraceptive reasons. Others considered that they had not experienced any pregnancy symptoms at all, suggesting that a lack of knowledge or self-awareness, denial or some other personal perceptions were influential. The shock and potential anxiety of conceiving outside of optimum conditions, for example an unplanned or unexpected pregnancy (the majority of women in this study), may have resulted in an inability to place pregnancy symptoms into what one author calls a *'meaningful whole’*[27] leading to misinterpretation and delay. Many women admitted that they had not put all their symptoms together to build a picture of themselves as pregnant, and as such had not created a pregnancy 'identity’.

### Planning the pregnancy

Lack of pregnancy planning or intent to become pregnant plays a significant part in delayed attendance for antenatal care [27], but this is usually discussed in the context of the fear and ambivalence women feel after a pregnancy is confirmed, rather than the initial recognition of pregnancy signs and symptoms. Numerous factors influence a woman’s perception of the likelihood of her conceiving. Contraceptive use and its influence on women’s acceptance of pregnancy has not been mentioned in other studies but was highly significant in this study. The shocked comments of the eight women using contraception suggest a combination of factors: a lack of knowledge about conception and the risks of pregnancy whilst using contraception, but more importantly a lack of belief in becoming pregnant.

Ambivalence, even when pregnancy was desired, was a common reaction amongst women to the discovery of an unplanned pregnancy, and has been identified in previous studies as influencing the initiation of antenatal care [27,28]. The lack of preparation for pregnancy found in the study not only affected women’s 'mindset’ and delayed confirmation of the pregnancy, but also led to feelings of fear, depression and ambivalence amongst some women, particularly related to the consequences of the pregnancy. This resulted in the denial, delaying and avoidant coping strategies evident in many other studies [29,30]. These feelings and behaviours were particularly apparent amongst those who had considered a termination or who felt most likely to be judged, such as teenagers and substance misusing women. However this was not a common theme, and was far less prevalent than in other studies. This may in part be due to the particular women who were prepared to be interviewed, but also the effect of being interviewed by a midwife, even one not involved in their care.

### Accepting the pregnancy

Other authors have discussed an in-between or liminal state where women are neither pregnant or 'unpregnant’, between pregnancy discovery and pregnancy acceptance, when women would make the pregnancy 'official’ and take action [27]. For some women in the study, failure to accept their pregnancies by refusing or ignoring a pregnancy test was clearly part of this 'little bit pregnant’ phase and demonstrated an avoidant coping strategy. For others, this phase lasted from days to months, whilst women considered whether to continue or terminate the pregnancy, and a process of passive continuation of the pregnancy ensued [31].

### The 'social pregnancy’

As other authors have observed, each pregnancy is a social phenomenon as well as a biological one and women need a certain amount of approval and social support before their pregnancy (and the need for antenatal care) can be acknowledged and accepted, both by the woman herself and her social network [27,29,32,33]. Access to antenatal care is heavily influenced by a woman’s willingness to embrace her pregnancy and particularly the social aspects of the pregnancy [17]. The dynamics between a pregnant woman and others influence both the 'discovery’ of a pregnancy and the creation of a woman’s pregnancy identity. At its most positive, such a supportive relationship can strongly influence the coping mechanisms of women, and reduce and prevent delay in accessing care. However where support was perceived to be lacking there was a reluctance to reveal the pregnancy, and thus to access care, for fear of disapproval, rejection, or 'consequences’.

In some cases in this study the resulting anxiety led to an initial denial and ongoing concealment, particularly from official confirmation and involvement, which continued for a significant proportion of the pregnancy, followed eventually by 'layers’ of revealing to those in their social network. This secrecy about pregnancy was particularly significant amongst young women, again a common theme in other studies [34,35].

This social network extends beyond family to community members and to potential care providers [29]. Lutz [36] discusses the idea of pregnancy as 'public life’: an external, idealised view of the woman’s life, pregnancy and family [35]. A pregnancy becomes public property once disclosed and made official, for example, by booking for antenatal care. As such, many women feel the need to assume a role, a positive image of themselves as capable, pregnant women, happy to be pregnant, which may not be the reality of the situation. Booking for care also crosses a line of inevitability, and demonstrates publicly a commitment to the pregnancy and to motherhood. As several women in the study indicated, fear of judgement, stigma, scrutiny or even the consequences of the pregnancy, such as coping with another child, meant that they were not ready to take this step, leading to avoidance or postponement of care. Women struggling to cope with difficult personal circumstances were particularly likely to delay access. However, many women simply had other priorities in their lives which impacted on their ability and willingness to engage with the 'public property’ of their pregnancy, and the accompanying care.

### Pregnancy as wellness

Many studies have identified that some women consider pregnancy a natural, normal life event, a state of 'wellness’ rather than a medical condition requiring immediate attention, and would only attend for care if unwell [18,33]. Similarly, in our study there was a fatalism towards or acceptance of the pregnancy, a positivism linked with a feeling of wellbeing, which led to some women either not trying to access early care or not challenging delays. In this way, a non-medicalised but very positive pregnancy identity also led to 'late booking’.

### Prioritising antenatal care

Other studies have demonstrated that beliefs about the importance of antenatal care are not always predictive of behaviour and do not account significantly for lack of use [37]. Almost all the women interviewed demonstrated some knowledge of antenatal care and support to attend it, and stated that early antenatal care was 'a good thing’. Many women demonstrated their understanding of the convention of attending for antenatal care, using expressions like, *“that’s what everyone does, isn’t it?” and “you need to go earlier, to see what’s correct”.* This was articulated more clearly than an understanding of the value or purpose of antenatal care, suggesting a wish to accord with a social norm rather than a rational and empirical belief in the importance of antenatal care.

For some women booking for antenatal care is an act of engagement with the maternity system, a system based on surveillance and prevention, which they may not subscribe to. However as we (and others) illustrate, most women accept the importance of antenatal care 'in theory’. For it to be acceptable 'in practice’ it needs to be appropriate, a good fit to the woman, an idea expressed elsewhere in healthcare research [18,38]. Women’s perceptions of convenience, and thus attendance, are influenced by their view of the *relevancy* of the care to themselves and their lives [37]. Our findings suggest that women made their own judgement of this 'fit’/relevance in relation to antenatal care, and their priorities reflected this. Dixon-Woods *et al’s*[39] idea of 'candidacy’ [38] is also reflected in the study, suggesting that access to healthcare is a dynamic process of negotiation, influenced by people engaging in defining their own understanding of what is appropriate medical attention and intervention for themselves. Some of the pregnant women in our study made this judgement in the context of their previous pregnancy experience, their beliefs and their acceptance of pregnancy. There was a suggestion amongst multiparous women that they were more relaxed about missing early care, because of their previous positive pregnancy experience, and that antenatal care was particularly important for first pregnancies, when women had more to learn. As in other studies, antenatal care was identified by many as important but not an immediate priority, something that could be postponed [18,29].

### Location of care

Though many lived in neighbourhoods with high deprivation indices and in low income households, a significant number of women in our study would not have been considered 'vulnerable’. Practical barriers to care have been widely reported in other studies [17,32-34], however only one of the women interviewed expressed any problems relating to their home circumstances or financial background that had prevented them from accessing care, again suggesting that most were well supported. The women’s responses suggested rather a differing set of priorities, a consideration of convenience and an assessment of the value of early care, linked to location, health and past experience. For example five women chose not to book for care until they were 'home’ - a place of perceived trust, familiarity and safety. All were able, but chose not, to access early care where they were, choosing other priorities in their lives. This illustrates how perceptions of the value and convenience of antenatal care in turn affect women’s attitudes towards its timing, portability and necessity, but also the importance women place on a familiar environment for such care. Women perceived their late attendance as an inconvenience or something to be expected, rather than feeling that they had been prevented from accessing care, reflecting women’s experience of actively choosing to delay care, from a considered and experienced perspective [40].

### Coping strategies

Where women had other priorities and had made the decision to avoid or postpone antenatal care, they demonstrated differing strategies to deal with their decision. For some women it was simply denial, a refusal to acknowledge publicly their pregnancy, a passive acceptance of delay or 'hoping for the best’ based on instinct *(“I just assumed I’d be all right”).* However, others demonstrated a more active, considered approach to their pregnancies, engaging in a process of self-care. Two women explicitly (and others implicitly) used their experience of pregnancy, both theoretical and real, to make sophisticated judgements about the progress of their pregnancies. This self-reliance was seen as a positive, purposeful thing by the women, who wished to take control, stay well and informed, and make their own decisions about the pregnancy.

Some authors have presented this self-care in negative terms as passive non-attendance based on ignorance [40,41]. Our study suggests a more positive view, women making what they perceived as the best choices themselves in their circumstances, when they feared this control and choice would be removed; the 'taking care of self’ suggested by Sword [37]. This presents women making decisions from a considered perspective, associated with the belief that pregnancy is a natural state that does not require early professional intervention [27,31,37]. This thoughtful process echoes that seen elsewhere in healthcare research, such as Pound *et al’s*[42] notion of 'resistance’ to medicine taking, with non-compliance not simply a passive failure but the result of active engagement and decision-making by patients, demonstrating ingenuity and energy. This concept of women challenging orthodoxy, in choosing to trust their own instincts about maintaining good health in pregnancy, requires further examination as it suggests a link to women’s health beliefs and/or a possible cynicism about the medicalisation of the 'normal’ process of pregnancy.

### Accepting delay

Some cases in the study highlighted the lack of a coherent approach between primary, community and secondary care towards the management of early pregnancy. A significant number of women in the study experienced administrative and professional failures, sometimes as a result of misdiagnosis of pregnancy or mis-estimation of gestation, leading to and exacerbating other delays. There is little evidence of this in other studies of late booking [18]. Most significant however was women’s acceptance and lack of challenge of these delays. On first examination this appeared to be because of a further lack of knowledge, particularly relating to the antenatal care 'system’. However this lack of knowledge was not a universal characteristic of the delayed women, as several were well educated about pregnancy and antenatal care. Women’s lack of empowerment and passive acceptance of the delays they encountered was linked to other significant factors, namely their 'mindset’ and preparedness for pregnancy, the trust they placed in healthcare professionals and the value and priority they placed on antenatal care, as a result of past experience, feeling well and the influence of others.

Overall the women’s reflections on their late booking have emerged as an additional area of interest from our study data. For example, the perception amongst some women that delayed access was a positive thing, with an impatience for the pregnancy to be over as soon as possible, has not previously been discussed. These findings reveal another layer of attitudes and behaviours influencing access, further pieces in the late booking 'jigsaw’, which merit further consideration.

## Conclusions

The timing of initial access to antenatal care is determined by a spectrum of decision-making, from acceptance through to a more passive non-acceptance and at the extreme an active rejection of the pregnancy and/or the need for antenatal care. This suggests a linear process, however the reality is often less structured, a combined 'web’ of these decisions bound up (in some cases) with preventative factors. This reflects the multiple interrelated influences on women’s acceptance of their pregnancies and their decision to access early antenatal care; a complex interaction of psychological, social and demographic factors which must be negotiated prior to a woman’s first antenatal appointment [19,43]. For a small number of (potentially the most vulnerable) women preventative factors may also be part of this acceptance and decision-making. Lack of reproductive knowledge could be part of this. To address this therefore requires intervention prior to conception as well as in early pregnancy.

The focus in this study has been the diversity of views of late booking women, in comparison to most previous studies which have targeted low income or vulnerable women from specific communities [13,17,19,29,35]. There are some resonant themes in our work with those previously published, such as lack of pregnancy recognition/planning, fear, chaos and self-care [18,27,29,31,37]. We found a lack of challenge to delays resulting from system and professional failures, and a complex relationship of beliefs and behaviours, combined with lack of reproductive knowledge, as the main threads running through the study. This lack of knowledge included knowledge of contraception, the symptoms of early pregnancy and the purpose, timing and value of antenatal care. It was most evident amongst nulliparous women and women with risk factors such as learning disabilities, substance misuse and for whom English was not their first language; often those identified as most at risk in previous maternal mortality reports [1,2]. Our group of study participants also included several women who might be considered 'low risk’ antenatally, had they not booked late. It may be that these women represent a larger group than previously identified. However, there is a lack of research relating to the socio-demographic characteristics of late booking women in the UK and if we are to challenge stereotypical categorisation this requires further study.

Lack of pregnancy planning has been linked to delayed access in previous studies and was evident in the majority of women in the study, some of whom were previous late bookers [27,29]. This, the existence of lay hindrance and a lack of active engagement in care suggests a need for improved promotion of the value and relevance of early antenatal care generally in the population. Pre-conceptual discussion/education is a key recommendation in the most recent UK maternal mortality report. However, as only an estimated 50% of pregnancies in the UK (and USA) are intended there is the need for opportunistic reproductive education and contraceptive counseling in community settings [2,44]. We consider that a more holistic view of women’s reproductive health (through women’s 'life course’) and reproductive health targeting need to be adopted. This would maximise opportunistic contraceptive/ health reviews in primary care, and re-emphasise the value of the 6 week postnatal check for women (currently poorly attended), to highlight the value and relevance of early antenatal care. Our study suggests that the risk of repeated late booking may be associated with direct and indirect experience of late booking, and this area is worthy of further research. There is also a role for community-based information/ advice campaigns (as recently introduced locally, influenced by the findings of this study) about early pregnancy symptoms and care, particularly targeting areas with higher than average late booking.

In addition, improved management of early antenatal care, the more 'joined up’ approach identified as missing by several women in the study, is required. Improved communication between community midwives, family doctors and reproductive and sexual health services, but also health and social care services that are outside of the National Health Service (NHS), could help to ensure the transfer of appropriate information, and the referral and follow up of women in early pregnancy. This could also help to ensure the prevention of system and professional failures.

Our study identified aspects of reproductive knowledge and beliefs relating to early pregnancy, as well as hindering and facilitating factors relating to lay and professional involvement. These require further investigation and analysis, in terms of what makes women present for pregnancy care, and the relationship between pregnant women and the professionals who care for them. The themes identified challenge over–simplistic perspectives concerning the reasons why women present late (socio-cultural adversity, the 'concealed pregnancy’, 'denial’) and the view of late bookers as passive and ignorant. Research to understand key practitioner perspectives on delayed access to antenatal care has been undertaken by the research team and will be published separately. Together we hope these studies will contribute to a greater understanding between pregnant women and health and social care practitioners in the future, and will be considered by both service commissioners and practitioners in order to promote the provision of timely antenatal care for all women.

## Abbreviations

UK: United Kingdom; USA: United States of America; DoH: Department of Health (UK); GP: General Practitioner (family doctor); G: Gravida; P: Parity; NHS: National Health Service (UK); USS: Ultrasound scan.

## Competing interests

The authors declare that they have no competing interests.

## Authors’ contributions

RH: Recruited the women and stakeholders, conducted the qualitative interviews, analysed the data and co-wrote the findings up for publication. GLJ: Conceived the original idea, wrote the protocol for the study, secured the funding for the study, secured ethical approval and research governance approval for the study, co-supervised the analysis of the data, including independent thematic verification and co-wrote the findings up for publication. CAM: Assisted with protocol development and recruitment of the stakeholders, co-supervised the analysis of the data, including independent thematic verification and co-wrote the findings up for publication. DOA: Conceived the original idea, assisted with protocol development and recruitment of the women and practitioners, co-supervised the analysis of the data and assisted with the writing of the manuscript. All authors read and approved the final manuscript.

## Authors’ information

R Haddrill BSc (Hons) MA PGCert *-* Postgraduate research student,^*,*^ School of Health and Related Research (ScHARR), University of Sheffield; Senior Lecturer in Midwifery, Sheffield Hallam University.

GL Jones BA (Hons), MA, D.Phil, - Reader in Social Science, Section of Health Economics & Decision Science, ScHARR, University of Sheffield.

CA Mitchell MBChB MD PGCert MEd - Senior Clinical Lecturer/General Practitioner, Academic Unit of Primary Medical Care, University of Sheffield.

DOC Anumba MBBS MD LL.M (Medical Law) - Professor of Obstetrics and Gynaecology and Honorary Consultant in Obstetrics & Fetomaternal medicine, Academic Unit of Reproductive and Developmental Medicine, University of Sheffield.

## Pre-publication history

The pre-publication history for this paper can be accessed here:

http://www.biomedcentral.com/1471-2393/14/207/prepub
